# Supplementary material for: Generalisable 3D printing error detection and correction via multi-head neural networks
Source: Nat Commun. 2022 Aug 15;13:4654. doi: 10.1038/s41467-022-31985-y (PMC9378646; doi:10.1038/s41467-022-31985-y)
Supplement: Supplementary file 1 — Supplementary Information [file 41467_2022_31985_MOESM1_ESM.pdf]

Supplementary Information

Generalisable 3D Printing Error Detection and Correction via Multi-Head Neural Networks

Douglas A. J. Brion[1,*], Sebastian W. Pattinson[1,*]

[1]Department of Engineering, University of Cambridge,
Trumpington Street, Cambridge, CB2 1PZ, UK

*Corresponding author. Email: dajb3@cam.ac.uk, swp29@cam.ac.uk

This PDF file includes:
Supplementary Figures S1 to S3.
Further details on the automated part remover.
Descriptions of Supplementary Movies S1 to S4.

Other Supplementary Information for this manuscript include the following:
Supplementary Movies S1 to S4.
Source data CSV files for plots in manuscript figures.

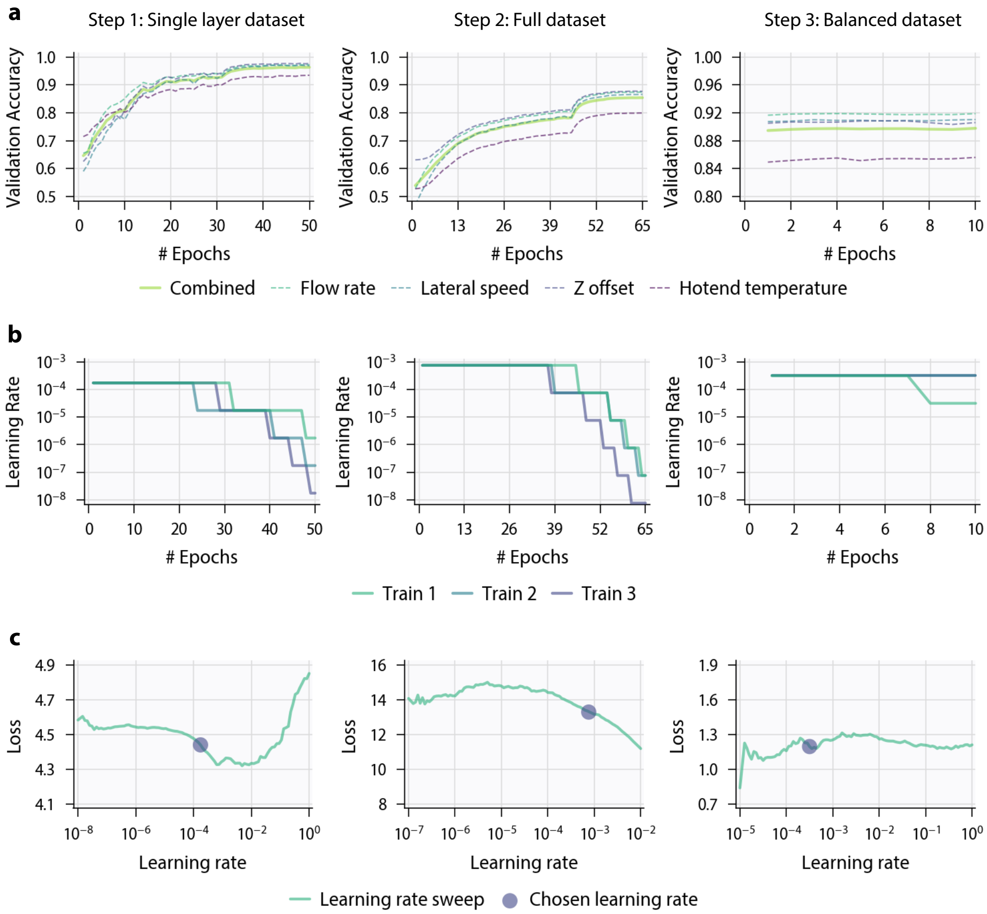

Fig. S1. Three stages of training the multi-head neural network with AM dataset. (**a**) Validation accuracy plots for each parameter and their combined mean for the best of 3 seeds, smoothed with an exponential moving average. (**b**) Learning rate decay for each train across 3 seeds using a reduce on plateau learning rate scheduler. Learning rate reduction results in noticeable increase in accuracy at certain points in (a). (**c**) Initial learning rate for each training stage was chosen by sweeping a wide learning rate range and selecting a value with a steep drop in loss.

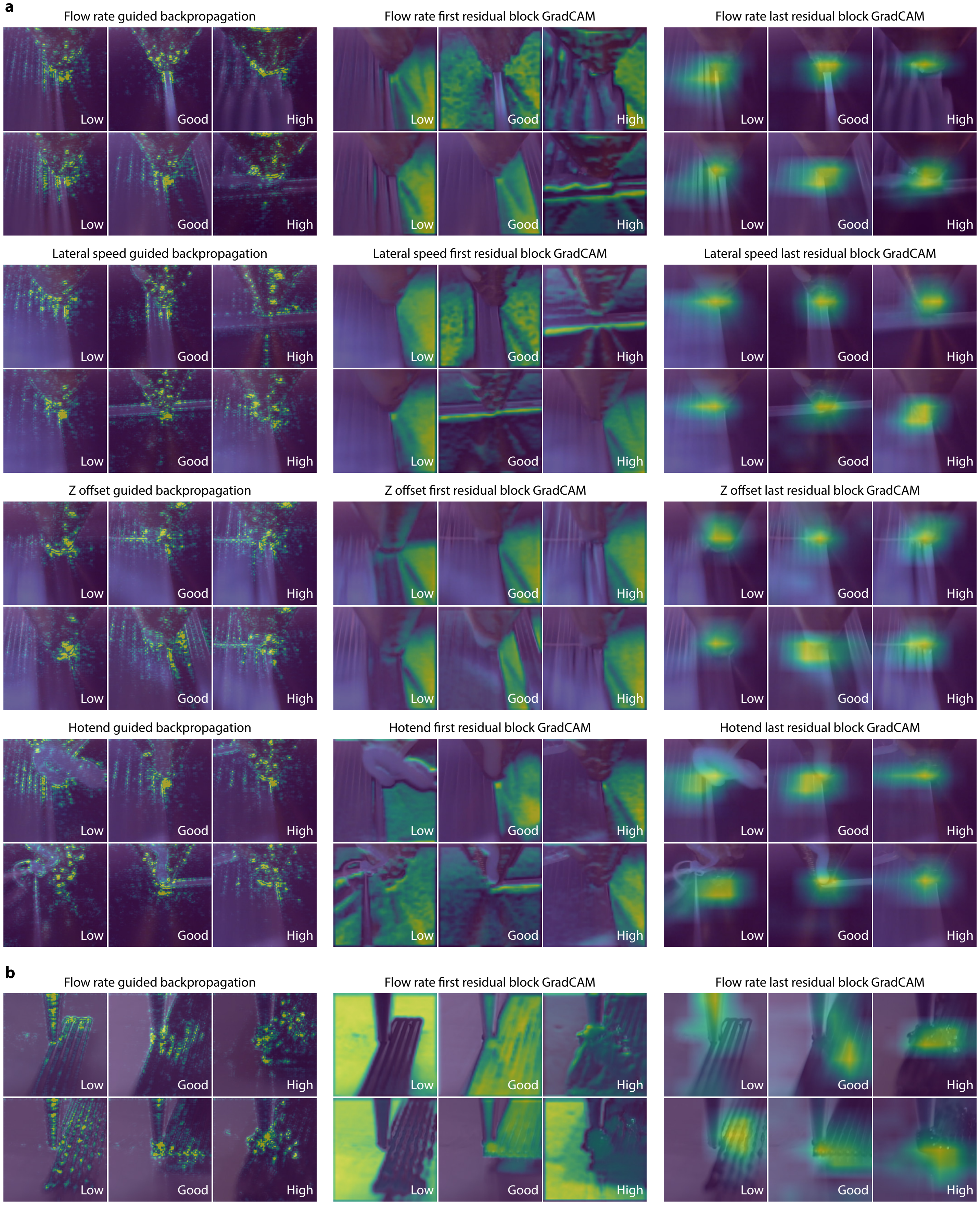
a
Flow rate guided backpropagation
Flow rate first residual block GradCAM
Flow rate last residual block GradCAM
Lateral speed guided backpropagation
Lateral speed first residual block GradCAM
Lateral speed last residual block GradCAM
Z offset guided backpropagation
Z offset first residual block GradCAM
Z offset last residual block GradCAM
Hotend guided backpropagation
Hotend first residual block GradCAM
Hotend last residual block GradCAM
b
Flow rate guided backpropagation
Flow rate first residual block GradCAM
Flow rate last residual block GradCAM
Low
Good
High

Fig. S2. Gradient-based saliency map visualisations of all classes and parameters. (**a**) Guided backpropagation and GradCAM applied across a set of 6 input images for each parameter. Representative examples chosen for low, good, and high classifications showing that similar features and regions are used for predictions. (**b**) Visualisations using same gradient methods on direct ink writing images to demonstrate that the network detects features and regions much like for thermoplastic extrusion.

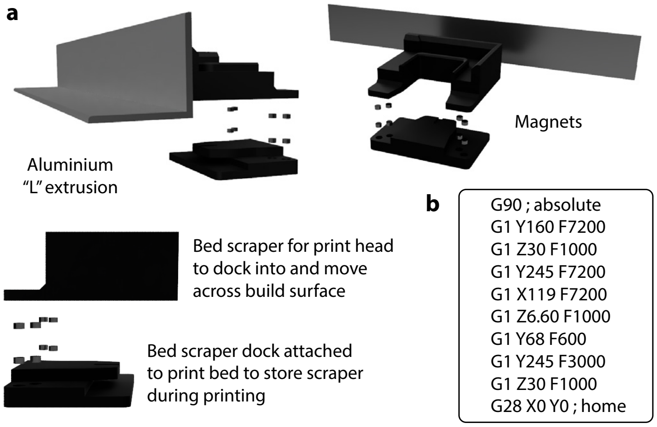

Fig. S3. Exploded diagrams of the part removal system and example G-code sequence for part removal procedure. (**a**) Three exploded diagrams of the complete bed remover system which can be easily added to existing printers. Magnets press fit into the printed bed scraper dock and bed scraper for the print head. In this work these parts were printed using black ABS filament. The aluminium "L" shaped extrusion is then attached either via glue or screw fastenings to the printed bed scraper. (**b**) Example G-code which can be run after printing to remove a part from the build surface. First, the absolute coordinate system is selected then a series of move commands are executed with the coordinates determined by the bed scraper's location and printer model.

Supplementary Note 1

Further details on the automated part remover. The removal sequence for the bed remover at the end of printing is as follows. First, there is a delay to wait for the hotend and bed temperatures to reach near room temperature. This minimises the risk of damage and helps reduce the adherence force between the printed part and build surface. Then the absolute coordinate system on the printer is selected in G-code and a series of moves are executed. The coordinates of these moves depend on the model of printer and the location of the bed scraper; however, the overall process is the same: move the print head to near the docked bed scraper, raise the Z height to avoid collision, move over the docked scraper in X and Y, slowly lower the print head into the bed scraper in the Z direction, drag the scraper using the print head across the entire build surface (the Y direction for this work) – this move should be slow, then return the scraper to its dock location, raise the print head out of the scraper in Z, and finally home

the printer. An example G-code sequence can be seen in fig. S3 for this process used on one of the Creality CR-20 Pro printers in this work.

Supplementary Movie S1.

Correction of each parameter individually (same prints as presented in fig. 4a). Clips show the predictions and actual parameters over time along with interventions during printing. Images of the print, cropped region, attention masks, and parameter specific GradCAM for the final layer are also shown.

Supplementary Movie S2.

Four videos showing the real-time multi-parameter discovery for different unseen thermoplastics (same prints as presented in fig. 4b). Each print is started with a different incorrect combination of flow rate, lateral speed, Z offset, and hotend temperature. All four parameters are simultaneously corrected using our system.

Supplementary Movie S3.

Guided backpropagation and GradCAM saliency maps for each of the single parameter correction prints (same prints as presented in fig. 4a).

Supplementary Movie S4.

A video showing the bed remover in operation, removing a printed part from the print bed.
