# Supplementary material for: Generalisable 3D printing error detection and correction via multi-head neural networks
Source: Nat Commun. 2022 Aug 15;13:4654. doi: 10.1038/s41467-022-31985-y (PMC9378646; doi:10.1038/s41467-022-31985-y)
Supplement: Supplementary file 3 — Description of Additional Supplementary Files [file 41467_2022_31985_MOESM3_ESM.pdf]

Description of Additional Supplementary Files

File Name: Supplementary Movie 1
Description: Correction of each parameter individually (same prints as presented in fig. 4a). Clips show the predictions and actual parameters over time along with interventions during printing. Images of the print, cropped region, attention masks, and parameter specific GradCAM for the final layer are also shown.

File Name: Supplementary Movie 2
Description: Four videos showing the real-time multi-parameter discovery for different unseen thermoplastics (same prints as presented in fig. 4b). Each print is started with a different incorrect combination of flow rate, lateral speed, Z offset, and hotend temperature. All four parameters are simultaneously corrected using our system.

File Name: Supplementary Movie 3
Description: Guided backpropagation and GradCAM saliency maps for each of the single parameter correction prints (same prints as presented in fig. 4a).

File Name: Supplementary Movie 4
Description: A video showing the bed remover in operation, removing a printed part from the print bed.
